# Oribatid mites (Acari, Oribatida) from riverine environments of some islands in Oceania

**DOI:** 10.3897/zookeys.318.5971

**Published:** 2013-07-24

**Authors:** Sergey G. Ermilov, Andrei V. Tolstikov, Nathalie Mary, Heinrich Schatz

**Affiliations:** 1 Tyumen State University, Tyumen, Russia; 2 Maharepa Moorea, French Polynesia; 3 University of Innsbruck, Institute of Zoology, Innsbruck, Austria

**Keywords:** Oribatida, riverine environment, checklist, new species, *Fortuynia*, Oceania

## Abstract

A checklist of identified oribatid mite taxa from riverine freshwater environments from six islands in Polynesia (New Caledonia, Tahiti, Moorea, Rurutu, Tubuai, Raiatea) is presented; 18 species, 16 genera and eight families were recorded. *Trhypochthoniellus longisetus* (Berlese, 1904) and *Trimalaconothrus albulus* Hammer, 1972 prevailed on distribution. *Fortuynia smiti* **sp. n.** (Fortuyniidae) is described from New Caledonia. The new speciesis morphologically most similar to *Fortuynia marina* Hammen, 1960 from New Guinea, but it differs from the latter by the longer notogastral setae *dm*, *lm*, *c*_2_, *p*_1_, epimeral setae *3b* and adanal setae *ad*_1_ and the presence of prodorsal lateral ridges.

## Introduction

At present, the fauna of oribatid mites (Acari: Oribatida) of the Oceania islands (Australian region) is studied insufficiently (for example: Jacot 1924, 1934; Sellnick 1959; Hammer 1971, 1972, 1973; Mahunka 1982; Balogh and Balogh 1986; Schabetsberger et al. 2009).

Our research is based on total oribatid mite material, which was collected by Nathalie Mary and Harry Smit from rivers of six islands of the Pacific region: New Caledonia (Melanesia), Tahiti, Moorea, Rurutu, Tubuai, Raiatea (all Polynesia). The primary purpose of this paper is to present a checklist of identified taxa.

In the course of taxonomic identification we found a new species, belonging to the genus *Fortuynia* Hammen, 1960 (Ameronothroidea, Fortuyniidae). The secondary purpose of the paper is to describe and illustrate this species under the name *Fortuynia smiti* sp. n. The genus *Fortuynia* is proposed by Hammen (1960) with *Fortuynia marina* Hammen, 1960 as type species. Currently, it comprises 10 species and two subspecies, which collectively are distributed in the Pantropical and Subtropical regions (sensu Subías 2004, updated 2013). The generic characters of *Fortuynia* were presented by Hammen (1960) and also summarized by Balogh and Balogh (1992) and Bayartogtokh et al. (2009). The identification keys to species of the genus have been presented earlier by Luxton (1986), Marshall and Pugh (2002) and Bayartogtokh et al. (2009).

## Materials and methods

The oribatid mite material was collected by Harry Smit and Nathalie Mary from several Pacific Islands. Smit’s oribatid mite material: all samples are water samples, made with a dip net. Mary’s oribatid mite material: all samples were taken with a surber net when sampled the benthos of the rivers and streams.

### List of localities

Melanesia: New Caledonia

01: Marais de la Rivière Blanche, Parc de la Rivière Bleue, 26.IX.2000, collected by H. Smit.

02: Koné Rivière, 10 km east of Koné, 01.X.2000, collected by H. Smit.

Polynesia – Society Islands: Tahiti

03: Papenoo River, 25.VI.2007, collected by N. Mary.

04: Vahiria River, 26.VI.2007, collected by N. Mary.

05: Vaitepiha River, 27.VI.2007, collected by N. Mary.

Polynesia – Society Islands: Moorea

06 Opunohu River, 24.VI.2007, collected by N. Mary.

07: Vaihana River, 07.VII.2007, collected by N. Mary.

08: Vaipapa River, 08.VII.2007, collected by N. Mary.

09: Paopao River, 09.VII.2007, collected by N. Mary.

Polynesia – Austral Islands: Rurutu

10: Vairee River, 30.VI.2007, collected by N. Mary.

11: Te Vaavai River, 01.VII.2007, collected by N. Mary.

12: Vaipapa River, 01.VII.2007, collected by N. Mary.

13: Peva Iti River, 02.VII.2007, collected by N. Mary.

Polynesia – Austral Islands: Tubuai

14: Vaitoaha River, 04.VII.2007, collected by N. Mary.

15: Matarahu River, 04.VII.2007, collected by N. Mary.

16: Hautara River, 05.VII.2007, collected by N. Mary.

17: Taahuaia River, 05.VII.2007, collected by N. Mary.

18: Vaiapu River, 05.VII.2007, collected by N. Mary.

Polynesia – Society Islands: Raiatea

19: Vaiatarau River, 11.VII.2007, collected by N. Mary.

20: Apoomau River, 11.VII.2007, collected by N. Mary.

21: Vaimariri River, 12.VII.2007, collected by N. Mary.

All specimens were studied in lactic acid, mounted in temporary cavity slides for the duration of the study, and then stored in 70% alcohol in vials. Body measurements are presented in micrometers. The body length was measured in lateral view, from the tip of the rostrum to the posterior edge of the ventral plate. Notogastral width refers to the maximum width in dorsal aspect. Lengths of body setae were measured in lateral aspect. Formula for leg setation are given in parentheses according to the sequence trochanter–femur–genu–tibia–tarsus (famulus included). Formula for leg solenidia are given in square brackets according to the sequence genu–tibia–tarsus. Terminology used in this paper mostly follows that summarized by Hammen (1960), Norton and Behan-Pelletier (2009).

### Faunistic aspect

We recorded 18 species belonging to 16 genera and eight families. *Ceratozetes hamobatoides* Hammer, 1967 is a new record for Oceania (previously known from New Zealand), all other taxa were recorded in Oceania previously. *Trhypochthoniellus longisetus* (Berlese, 1904) and *Trimalaconothrus albulus* Hammer, 1972 prevailed on distribution (found in 19 localities on six and five islands, respectively). Also, *Hydrozetes lemnae* (Coggi, 1897) is registered from 11 localities (on five islands), *Scheloribates praeincisus* (Berlese, 1910) in six localities (on three islands). The majority of species (13 from 18) were found in 1–3 localities (Table 1). Comparing a previous investigation of oribatid mites in freshwater on Pacific islands (Schabetsberger et al. 2009) three species found in lentic waters of Pacific islands are common with records of the present study (*Hydrozetes lemnae* – Fiji, *Nasozetes stunkardi* Sengbusch, 1957 – Fiji, *Trhypochthoniellus longisetus* – Samoa).

**Table 1.** List and distributions of identified taxa from the Oceania islands (distribution data from Subías 2004, updated and actualized. Ecoregions according to Olson et al. 2001). Islands: NC – New Caledonia, Ta – Tahiti, Mo – Moorea, Ru – Rurutu, Tu – Tubuai, Ra – Raiatea.

**Taxa**	**Distributions of species**	**NC**		**Ta**			**Mo**				**Ru**				**Tu**					**Ra**		
		**01**	**02**	**03**	**04**	**05**	**06**	**07**	**08**	**09**	**10**	**11**	**12**	**13**	**14**	**15**	**16**	**17**	**18**	**19**	**20**	**21**
Lohmanniidae																						
*Ozacarus tahitiensis* (Hammer, 1972)	Oceania – Polynesia (Tahiti, Moorea)	-	-	-	-	-	-	-	+	-	-	-	-	-	-	-	-	-	-	-	-	-
Trhypochthoniidae																						
*Archegozetes magnus* (Sellnick, 1925)	Afrotropic, Neotropic, Indo-Malay, Australasia (New Guinea), Oceania – Polynesia (Tonga, Eua, Moorea)	-	-	-	-	+	-	-	+	-	-	-	-	-	-	-	-	-	-	-	-	-
*Trhypochthoniellus longisetus* (Berlese, 1904)	Holarctic, Afrotropic, Neotropic, Indo-Malay, Australasia (Australia, New Zealand), Oceania – Melanesia (New Caledonia), Polynesia (Hawaii, Samoa, Tahiti, Moorea, Rurutu, Tubuai, Raiatea)	-	+	+	+	+	+	+	+	+	+	+	+	+	-	+	+	+	+	+	+	+
Malaconothridae																						
*Trimalaconothrus albulus* Hammer, 1972	Indo-Malay (Taiwan). Oceania – Polynesia (Tahiti)	-	-	+	+	+	+	+	+	+	+	+	+	+	+	+	+	+	+	+	+	+
Nanhermanniidae																						
*Masthermannia* sp.	–	-	-	-	-	+	-	-	-	-	-	-	-	+	-	-	-	-	-	-	-	+
Hermanniidae																						
*Phyllhermannia pacifica* (Hammer, 1972)	Oceania – Polynesia (Tongatapu, Eua, Tahiti, Rangiroa, Tubuai)	-	-	-	-	-	-	-	-	-	-	-	-	-	-	+	-	-	-	-	-	-
Hydrozetidae																						
*Hydrozetes lemnae* (Coggi, 1897)	Palaearctic, Afrotropic, Neotropic (also Galapagos islands), Indo-Malay (Indonesia, Philippines), Australasia (Australia, New Zealand), Oceania – Melanesia (New Caledonia), Polynesia (Fiji, Upolu, Tahiti, Moorea, Rurutu, Tubuai, Raiatea, Easter Island)	+	-	+	+	+	+	-	+	+	-	-	-	-	-	-	+	+	+	-	-	+
Fortuyniidae																						
*Fortuynia smiti* sp. n.	Oceania – Melanesia (New Caledonia)	-	+	-	-	-	-	-	-	-	-	-	-	-	-	-	-	-	-	-	-	-
Ceratozetidae																						
*Ceratozetes hamobatoides* Hammer, 1967	Australasia (New Zealand), Oceania – Melanesia (New Caledonia)	+	-	-	-	-	-	-	-	-	-	-	-	-	-	-	-	-	-	-	-	-
Humerobatidae																						
*Humerobates rostrolamellatus* Grandjean, 1936	Holarctic, Afrotropic, Neotropic, Indo-Malay (Okinawa), Oceania – Polynesia (Eua, Moorea, Hawaii)	-	-	-	-	-	+	-	-	-	-	-	-	-	-	-	-	-	-	-	-	-
Scheloribatidae																						
*Nasozetes stunkardi* Sengbusch, 1957	Indo-Malay (Philippines), Oceania – Micronesia (Guam), Polynesia (Fiji, Raiatea)	-	-	-	-	-	-	-	-	-	-	-	-	-	-	-	-	-	-	-	+	-
*Tuberemaeus indentatus* (Hammer, 1973)	Oceania - Polynesia (Upolu, Raiatea)	-	-	-	-	-	-	-	-	-	-	-	-	-	-	-	-	-	-	-	+	-
*Scheloribates praeincisus* (Berlese, 1910)	Neotropic (Panama, Brazil, Galapagos islands) Indo-Malay (India, Indonesia, Philippines). Oceania – Polynesia (Fiji, Tonga, Eua, Upolu, Tahiti, Moorea, Borabora, Rangiroa, Rurutu, Raiatea)	-	-	-	-	-	-	-	+	-	+	-	-	+	-	-	-	-	-	+	+	+
*Scheloribates tubuaiensis* Sellnick, 1959	Palaearctic (Caucasus), Oceania – Polynesia (Tongatapu, Upolu, Moorea, Tubuai, Rurutu)	-	-	-	-	-	-	+	+	+	+	-	-	-	-	-	+	-	-	-	-	-
Oripodidae																						
*Benoibates marginatus* (Hammer, 1973)	Oceania – Polynesia (Eua, Raiatea)	-	-	-	-	-	-	-	-	-	-	-	-	-	-	-	-	-	-	-	-	+
Haplozetidae																						
*Protoribates bipilus* (Hammer, 1972)	Oceania – Polynesia (Tongatapu. Tahiti, Rurutu, Tubuai)	-	-	-	-	-	-	-	-	-	+	-	-	-	-	-	-	+	-	-	-	-
Galumnidae																						
*Galumna euaensis* Hammer, 1973	Oceania – Polynesia (Eua, Moorea, Raiatea)	-	-	-	-	-	-	-	+	+	-	-	-	-	-	-	-	-	-	-	+	-
*Galumna valida* Aoki, 1994	Oceania – Micronesia (Marianas: Maug islands), Polynesia (Moorea)	-	-	-	-	-	-	-	+	+	-	-	-	-	-	-	-	-	-	-	-	-

## Description of new species

### Fortuynia smiti

Ermilov, Tolstikov, Mary & Schatz sp. n.

urn:lsid:zoobank.org:act:7400FB31-6262-4525-A1A5-AD92C8696501

http://species-id.net/wiki/Fortuynia_smiti

Figs 1 – 13

#### Diagnosis.

Body size 564–614 × 381–431. Body surface microfoveolate. Lamellar lines, internal and lateral ridges developed. Rostral setae weakly thickened, with short cilia; lamellar setae thin, slightly barbed. Interlamellar and exobothridial setae minute. Sensilli short, clavate, smooth. Notogaster with 14 pairs of setae and one pair of setal alveoli (*c*_3_). Length of setae *c*_1_, *da* > *c*_2_, *dm*, *la*, *lm* > *p*_1_ > *lp*, *h*_3_ > *dp*, *h*_1_, *h*_2_ > *p*_1_, *p*_2_. Adanal setae *ad*_1_ longer than other adanal setae.

#### Description.

**Male.** *Measurements*. Body length 581 (holotype, male), 564–614 (six paratypes, all males); body width 398 (holotype), 381–431 (six paratypes, all males).

*Integument*. Body color brown to yellow-brownish. Body surface microfoveolate (clearly visible under high magnification, ×1000). Lateral podosomal regions with tuberculate cerotegument (diameter of tubercles up to 6).

*Prodorsum*. Rostrum rounded. Lamellar lines (*ce*) strong, equal to half of prodorsum. Internal ridges (*ci*) present, very thin, reaching insertions of interlamellar setae. Anterior part of lamellar lines with short lateral ridges (*cl*), which are located perpendicularly to them. Rostral setae (*ro*, 69–82) setiform, weakly thickened, with short cilia, set on small tubercles. Lamellar setae (*le*, 41–45) setiform, thin, slightly barbed. Interlamellar (*in*) and exobothridial (*ex*) setae minute (1), poorly visible. Sensilli (*ss*, 32–36) curved backwards, with short stalk and longer clavate, smooth head.

*Notogaster*. Lenticulus present, with amorphic borders. Notogastral region with 14 pairs of setiform, smooth notogastral setae and one pair of setal alveoli (*c*_3_). Setae *c*_1_, *da* (90–102) longer than *c*_2_, *dm*, *la*, *lm* (69–82), *p*_1_ (30–36), *lp*, *h*_3_ (28–32), *dp*, *h*_1_, *h*_2_ (20–24); *p*_1_, *p*_2_ shortest (4–6). Lyrifissures *ia*, *im*, *ip*, *ih* and *ips* and opisthonotal gland openings (*gla*) distinct, located typically for the genus.

*Gnathosoma*. Subcapitulum longer than wide (118–205 × 143–151). Subcapitular setae setiform, smooth; *h* (82–86) longer than *a* and *m* (both 53–57). Lips only with one spiniform seta (*or*, 10–12). Palps (123–131) with setation 0–2–1–3–9(+ω). All setae smooth. Solenidion weakly thickened, blunt-ended, pressed to surface of tarsus, not attached with eupathidium. Chelicerae (188–205) with two barbed setae; *cha* (69–73) longer than *chb* (45–49). Trägårdh’s organ (Tg) long, conical.

*Epimeral and lateral podosomal regions*. Apodemes 1, 2, 3 and sejugal well developed. Apodemes 2 and sejugal fused medially. Epimeral setal formula 3–1–3–2; setae setiform, smooth. Setae *1b* (49–61) longer than *3b* (41–49), *1a*, *2a*, *3a*, *3c*, *4a*, *4c* (24–36); *1c* shortest (16–20). Pedotecta I (Pd I) of medium size, concave.

*Anogenital region*. Anogenital setae setiform, thin, smooth. Five pairs of genital (*g*_1_–*g*_5_), one pair of aggenital (*ag*), two pairs of anal (*an*_1_, *an*_2_) and the anterior two pairs of adanal (*ad*_2_, *ad*_3_) setae similar in length (28–32); only the first pair of adanal setae *ad*_1_ longer (41–53). Lyrifissures *iad* located in paraanal position.

*Legs*. Claw of each leg large, smooth. Porose areas developed typically for the genus (Bayartogtokh et al. (2009). Formulae of leg setation and solenidia: I (1–4–2–3–18) [1–2–2], II (1–4–2–3–15) [1–1–2], III (2–3–1–3–15) [1–1–0], IV (1–2–2–3–12) [0–1–0]; homology of setae and solenidia indicated in Table 2. Setae setiform, well or slightly barbed except smooth *p* on tarsi I, II and *s*. Famulus (*e*) short, straight, weakly blunt-ended. All solenidia long setiform, pointed.

**Table 2.** Leg setation and solenidia of adult *Fortuynia smiti* sp. n.

**Leg**	**Trochanter**	**Femur**	**Genu**	**Tibia**	**Tarsus**
I	*v*’	*d, (l), bv*’’	*(l)*, σ	*(l), v*’, φ_1_, φ_2_	*(ft)*, *(tc)*, *(it)*, *(p)*, *(u)*, *(a)*, *s*, *(pv)*, *(pl)*, *e*, ω_1_, ω_2_
II	*v*’	*d, (l)*, *bv*’’	*(l)*, σ	*(l), v*’, φ	*(ft), (tc), (it), (p)*, *(u), (a), s, (pv)*, ω_1_, ω_2_
III	*l’, v*’	*d, l’, ev*’	*l*’, σ	*l’, (v)*, φ	*(ft), (tc), (it), (p)*, *(u), (a), s, (pv)*
IV	*v*’	*d, ev*’	*d, l*’	*l’, (v)*, φ	*ft’’, (tc), (p)*, *(u), (a), s, (pv)*

Roman letters refer to normal setae (*e* to famulus), Greek letters to solenidia. Single prime (’) marks setae on anterior and double prime (’’) setae on posterior side of the given leg segment. Parentheses refer to a pseudosymmetrical pair of setae.

**Figures 1–3.**
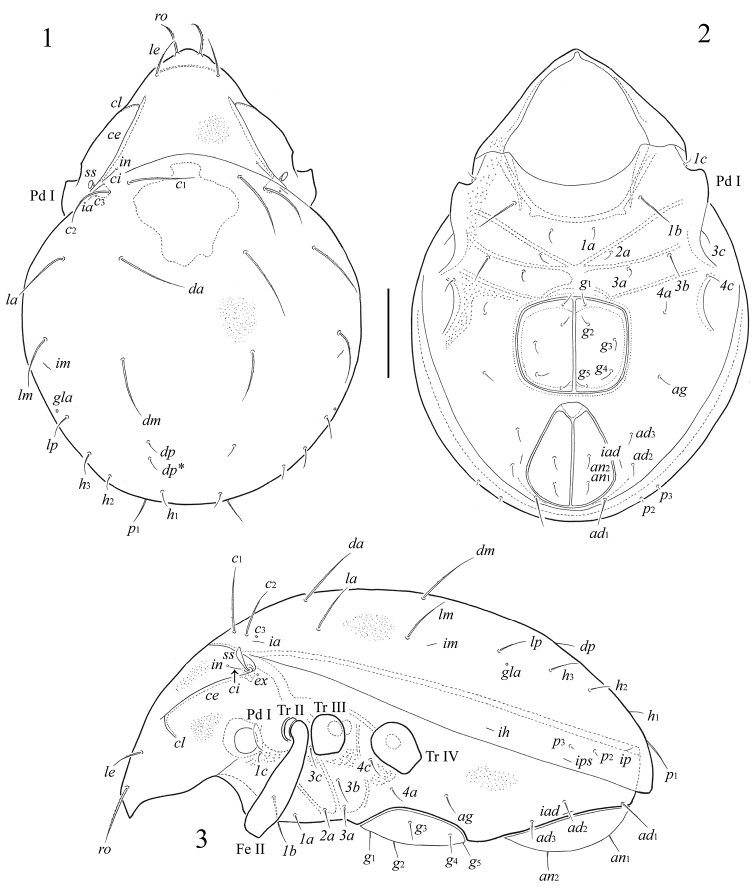
*Fortuynia smiti* sp. n., adult: **1** dorsal view **2** ventral view (gnathosoma and legs not illustrated) **3** lateral view (gnathosoma and legs except femur II and trochanters II–IV not illustrated). Scale bar 100 μm.

**Figures 4–11:**
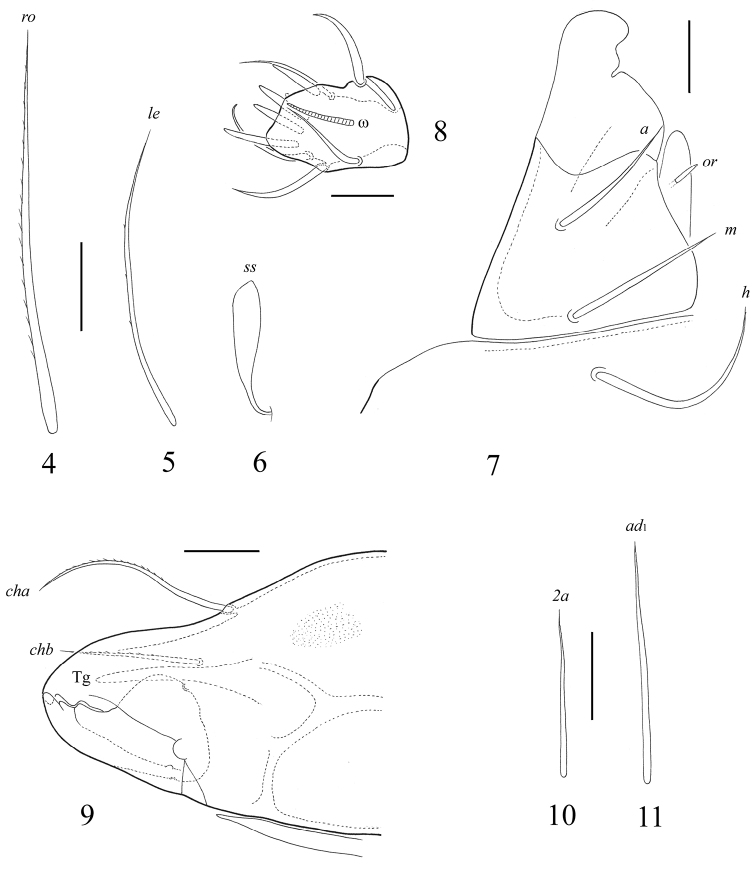
. *Fortuynia smiti* sp. n., adult: **4** rostral seta **5** lamellar seta **6** sensillus **7** subcapitulum, right half of anterior part, ventro-lateral view **8** palptarsus **9** chelicera, anterior part **10** epimeral seta *2a* **11** adanal setae *ad*_1_. Scale bar (**4–7**, **9–11**) 20 μm, (8) 10 μm.

**Figures 12–13:**
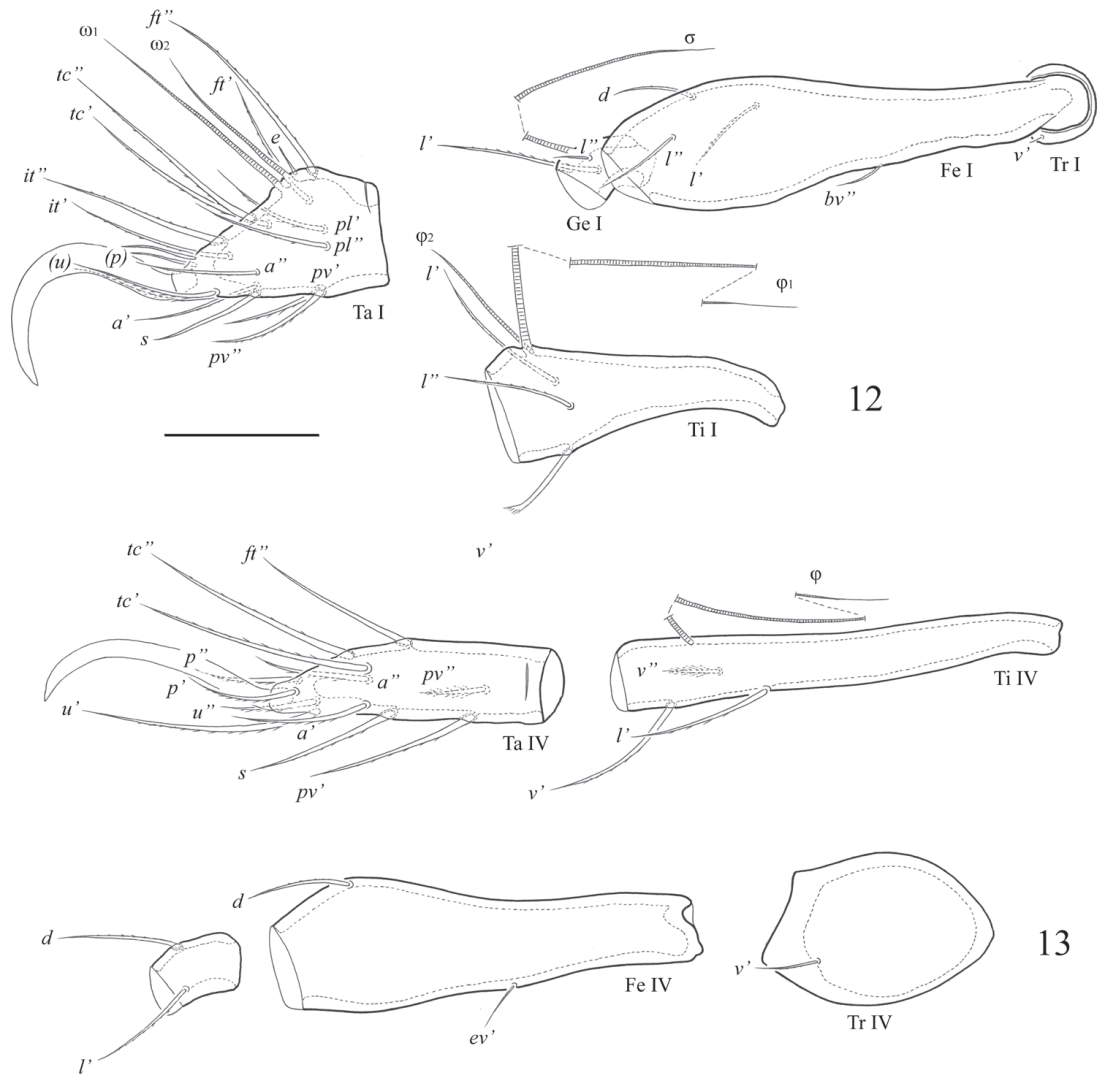
. *Fortuynia smiti* sp. n., adult: **12** segments of leg I, left, antiaxial view **13** segments of leg IV, right, antiaxial view. Scale bar 50 μm.

#### Material examined.

Holotype (male) and six paratypes (all males): Locality 02.

#### Type deposition.

The holotype (in alcohol) is deposited in the collection of the Zoological Institute of the Russian Academy of Sciences, St. Petersburg, Russia; two paratypes (in alcohol) are deposited in the collection of the Siberian Zoological Museum, Novosibirsk, Russia; four paratypes (in alcohol) are deposited in the collection of the Tyumen State University Museum of Zoology, Tyumen, Russia.

**Etymology.** The species is named after our colleague, the renowned acarologist, Dr. Harry Smit (Netherlands Centre for Biodiversity Naturalis, Leiden, The Netherlands), who has collected the specimens of *Fortuynia smiti* sp. n.

#### Comparison.

*Fortuynia smiti* sp. n. is most similar to *Fortuynia marina* Hammen, 1960 from New Guinea (Hammen 1960) in having following combination of morphological characters: body of medium size; presence of lamellar lines and internal ridges; presence of alveoli notogastral setae *c*_3_. However it differs from the latter by the long notogastral setae *dm*, *lm*, *c*_2_, *p*_1_, epimeral setae *3b* and adanal setae *ad*_1_ (versus short in *Fortuynia marina*) and the presence of prodorsal lateral ridges (versus absent in *Fortuynia marina*).

## Supplementary Material

XML Treatment for Fortuynia smiti

## Appendix 1

Figure 1

## Appendix 2

Figure 2

## Appendix 3

Figure 3
